# A Caregiver Survey of Driving Readiness in Teens Born Extremely Preterm

**DOI:** 10.1016/j.focus.2025.100405

**Published:** 2025-08-05

**Authors:** Rebecca J. McAdams, Jessica Quach, Dominique M. Rose, Blake Hardin, Carl Backes, Jingzhen Yang, H. Gerry Taylor

**Affiliations:** 1 Center for Injury Research and Policy, The Abigail Wexner Research Institute at Nationwide Children’s Hospital, Columbus, Ohio; 2 Center for Biobehavioral Health, The Abigail Wexner Research Institute at Nationwide Children’s Hospital, Columbus, Ohio; 3 Center for Perinatal Research, The Abigail Wexner Research Institute at Nationwide Children’s Hospital, Columbus, Ohio; 4 Department of Pediatrics, The Ohio State University College of Medicine, Columbus, Ohio; 5 Division of Epidemiology, The Ohio State University College of Public Health, Columbus, Ohio

**Keywords:** Teen driving, driving readiness, preterm birth

## Abstract

**Introduction:**

Teens born extremely preterm (defined as a gestational age of <28 weeks) are at a higher risk of cognitive and physical disabilities, potentially affecting their ability to learn to drive safely. This exploratory study surveyed caregivers to obtain their perceptions of driving readiness in teens born extremely preterm.

**Methods:**

A cross-sectional survey of caregivers of teens (aged 14–18 years) residing in Ohio who were born extremely preterm was conducted to assess teen interest and progress in obtaining a driver’s license, reasons for their lack of progress, and caregiver involvement in helping their teen to learn to drive.

**Results:**

The sample included 60 caregivers of teens distributed across 4 age groups based on license eligibility. Most teens (87%) had a history of a neurodevelopmental or medical disorder. Only 9 (30%) of 30 teens aged <18 years who were age-eligible for a temporary permit had obtained one, and only 1 (7%) of 14 teens who was age-eligible for a probationary license had obtained one. Rates of neurological disorders and concerns that the teen lacked maturity to drive or would get lost were significantly higher in 25 age-eligible teens who were not driving than in the 20 teens who were driving (*p*=0.013 and 0.038, respectively). Caregivers frequently talked to their teens about driving but less often practiced driving with their teens.

**Conclusions:**

Delays in progressing toward obtaining a driving license are common in teens born extremely preterm. The findings from this study describe these delays and provide information on caregiver concerns and involvement in driving. Future research is needed to characterize and improve driving readiness in this population.

## Highlights

• Caregiver survey shows that only few teens born extremely preterm have driver’s licenses.
• Survey also reveals a low rate of obtaining temporary permits among these teens.
• Majority of these teens have caregiver-reported physical or developmental problems.
• Caregiver concerns about driving were more among age-eligible teens who were not driving.
• These study findings emphasize the need to improve driving readiness among these teens.

## Graphical Abstract

**Figure:**
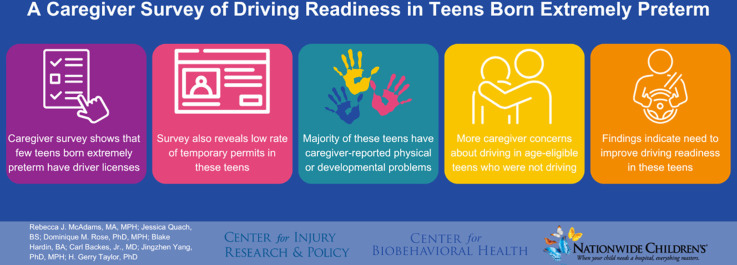

## INTRODUCTION

Motor vehicle collisions (MVC) are the leading cause of death among U.S. teens, particularly during their first few months of unsupervised driving.^1^, ^2^, ^3^, ^4^, ^5^ Contributing factors include underdeveloped driving skills and limited experience in navigating challenging driving situations such as adapting to varying road conditions, driving at night, and anticipating potential hazards.^6^, ^7^, ^8^, ^9^, ^10^, ^11^ Since the 1990s, all U.S. states and the District of Columbia have enacted the graduated driver licensing (GDL) laws to reduce risks associated with teen driving by ensuring new drivers gain supervised experience and limited exposure to risky situations.^12^ Although MVC rates in teens were reduced following the adoption of these laws,^12^, ^13^, ^14^ teen drivers continue to be at the highest risk for collisions and associated fatalities among all age groups.^5^

Teens with developmental disabilities may be less ready to drive safely than their typically developing peers. Compared to other youth, teens with attention-deficit/hyperactivity disorder (ADHD) have higher rates of collisions^15^, ^16^, ^17^, ^18^, ^19^ and young drivers with autism spectrum disorder (ASD) have more driving citations and weaknesses in driving-related skills, such as recognizing road hazards, slower response speed, and poorer inhibitory control and situational awareness.^20^, ^21^, ^22^ Additional research has documented the associations of weaknesses in cognitive development with risks for MVC, difficulties in learning to drive among youth with conditions such as cerebral palsy and spina bifida, and problems in resuming driving among youth who have sustained a traumatic brain injury.^23^, ^24^, ^25^

However, the authors did not find any studies on the driving readiness of teens born preterm (gestational age [GA] of <37 weeks), who comprise approximately 10% of youth.^26^ Deficits in motor abilities and cognitive skills are evident across the full spectrum of preterm birth, but they are most pronounced in youth born extremely preterm (EP, GA of <28 weeks).^27^, ^28^, ^29^, ^30^, ^31^ Some of these deficits, including slow response speed and problems in attention, executive function, and motor coordination, could impair teens’ driving skills and increase their risk of collisions.

Consequently, the authors hypothesize that the caregivers of teens born EP would have multiple concerns about their driving and would report limited interest in or progression toward licensure for their teens. Findings confirming this hypothesis would imply that many of these youth may not be taking advantage of opportunities to practice driving as part of the GDL process (prior to age 18 years), potentially hindering their independence and social integration with peers.^3^ Specific study aims were to examine caregiver-reported perceptions of: (1) the levels of interest in and progress toward licensure for teens aged 14–18 years who were born EP, (2) reasons for the lack of progress in obtaining a temporary permit or license and caregiver concerns regarding teen driving, and (3) involvement in helping their teens learn to drive.

## METHODS

### Study Sample

Caregivers were eligible for this study if they were proficient in reading English, aged ≥18 years, current Ohio residents, and primary caregiver of a teen born EP and aged 14–18 years at the time of the survey. Focus on teens born EP was justified by findings documenting that most of these youth have medical and developmental disorders,^32^ representing the subset of youth born preterm most likely to have problems in participating in the GDL process. Teen sex was defined as sex at birth. Surveys were completed between April 2024 and August 2024 by the caregiver for only 1 teen per family. Exclusions included nonlegal guardians of the teen and teens who were wards of the state. Caregivers completed a HIPAA-compliant Research Electronic Data Capture (REDCap) survey tailored to their teen’s age group. Group 1 comprised teens not yet eligible to drive (ages, 14 years 0 months to 15 years 5 months), Group 2 comprised teens eligible for only a temporary permit (ages, 15 years 6 months to 15 years 11 months), Group 3 comprised teens eligible for a probationary license (ages, 16 years 0 months to 17 years 11 months), and Group 4 comprised teens eligible for the unrestricted Ohio driver’s license (ages, 18 years 0 months to 18 years 11 months). Given an interest in surveying readiness at various stages of licensing eligibility and the exploratory nature of the study, sample size was based on practical considerations rather than on estimates of statistical power. Resource limitations also precluded the collection of teen self-reports or recruitment of caregivers of term-born comparison teens.

The study sample was limited to caregivers of teens born EP, cared for as neonates at a single children’s hospital in the Midwestern U.S., and identified via a medical record search. Only the teen’s name, date of birth, sex at birth, family address, and phone number were accessed. Recruitment goals were to obtain ∼15 caregiver surveys in each of the 4 driving stage groups. Recruitment began with a mailed letter containing a QR code and survey link. Caregivers who did not respond within 10 days received a follow-up call from the study team; voicemails were left if unanswered. Caregivers who declined, did not respond to the letter or calls after 3 attempts, or had not completed the survey were replaced with another potential caregiver. Thirteen of 240 caregivers completed the consent and survey after receiving the recruitment letter, and 47 additional caregivers did so after follow-up calls, leading to a total of 60 participants. The study was approved by the authors’ IRB.

### Measures

The survey asked about caregiver demographics, along with a caregiver report of teen physical or developmental disorders. Caregivers were then asked about their teen’s interest in driving, their willingness to allow their teen to work toward licensure, reasons for not progressing toward licensure, and concerns regarding their teen’s driving. Dates that the teens attained their temporary permit, probationary license, or full Ohio driver’s license were obtained via parent report and used to calculate the age at attainment based on the time between these dates and the teen’s birthdate. Caregivers of the teens in Groups 2–4 who were driving also rated the frequency with which they were involved in instructing their teens to drive. The frequencies of each of 3 types of involvement (talked about, showed, and practiced driving) were rated on a scale with 1=*never*, 2=*rarely*, 3=*infrequently*, 4=*frequently*, and 5=*very frequently*. Although the survey was developed for this study, potential parent concerns and levels of involvement were based on findings from previous research on teen driving.^8^^,^^33^, ^34^, ^35^, ^36^, ^37^

### Statistical Analysis

The data were analyzed using SAS version 9.4.^38^ Descriptive statistics described caregiver characteristics, using frequencies and percentages for categorical variables and mean and SDs for continuous variables. A dichotomous variable was created to identify teens with neurological, chronic non-neurological, vision or hearing, and developmental disorders. Chi-square or Fisher’s exact tests were conducted to examine the associations of driving status with caregiver concerns and physical and developmental disorders. All 60 caregivers completed the survey, with no missing data and significance defined as *p*>0.05.

## RESULTS

Teens of the 60 participating caregivers included 15 in Group 1 (including 10 males), 16 in Group 2 (including 8 males), 14 in Group 3 (including 9 males), and 15 in Group 4 (including 9 males). Caregiver demographic characteristics are listed in Table 1. Fifty-two (87%) teens had at least 1 of 4 types of physical or developmental disorders, including neurological disorders such as cerebral palsy, epilepsy, and traumatic brain injury (*n*=20, 33%); chronic non-neurological medical disorders such as asthma, diabetes, and Crohn disease (*n*=14, 23%); vision or hearing disorders (*n*=13, 22%); and developmental disorders such as ADHD, ASD, learning disabilities, and anxiety (*n*=46, 77%). Many teens had multiple disorders.

**Table 1:** Caregiver Demographic Characteristics

Characteristic	Overall,*n* (%) (N=60)	Age group, *n* (%)			
		Group 1 (*n*=15)	Group 2 (*n*=16)	Group 3 (*n*=14)	Group 4 (*n*=15)
Caregiver sex: Female	24 (40%)	5 (33%)	8 (50%)	5 (36%)	9 (40%)
Married	37 (62%)	9 (60%)	7 (44%)	8 (57%)	13 (87%)
Race/ethnicity					
White	44 (73%)	11 (73%)	11 (69%)	9 (64%)	13 (87%)
Black	13 (22%)	3 (20%)	4 (25%)	4 (29%)	2 (13%)
Other^a^	1 (2%)	1 (7%)	1 (6%)	1 (7%)	0 (0%)
Non-Hispanic	58 (88%)	15 (100%)	16 (100%)	12 (86%)	15 (100%)
Caregiver education					
High school or less	9 (15%)	4 (27%)	2 (13%)	2 (14%)	1 (7%)
Some college or bachelor’s degree	31 (52%)	8 (52%)	7 (44%)	6 (43%)	10 (67%)
Some graduate or professional degree	14 (23%)	2 (13%)	4 (25%)	4 (29%)	4 (27%)
Caregiver employed full-time	38 (63%)	9 (60%)	9 (56%)	9 (64%)	11 (73%)
Income >$150,000	17 (28%)	3 (20%)	3 (19%)	3 (21%)	8 (53%)
Caregiver is birth parent	53 (88%)	14 (93%)	12 (75%)	12 (85%)	15 (100%)

a One family was Asian and 2 families declined reporting race.

Caregivers reported that 9 (including 5 males) of the 15 teens who were not yet eligible to drive (Group 1; 60%) were interested in getting their temporary permit, 5 (all males, 33%) were not interested, and 1 male (7%) was unsure. All caregivers of the interested teens indicated that they would allow their teens to obtain a temporary permit. The 5 caregivers of the uninterested teens cited disability, such as blindness or an intellectual disability, as the reason for their teens’ disinterest.

The driving status of the 45 teens (26 males) who were age-eligible to drive (Groups 2–4) is summarized in Table 2. Twenty (10 males, 44%) of these teens were driving. Only 9 teens in Groups 2 and 3 (30%) were driving, including 4 (25%) in Group 2 and 5 (36%) in Group 3. The mean age at which the 9 teens obtained their temporary permit was 15 years 11 months (SD=9 months). Only 1 (7%) of those eligible for a probationary license (Group 3) had it. In contrast, 10 (including 5 males) of the teens in Group 4 (67%) had an Ohio driver’s license, a licensing rate significantly higher than that for Group 3 (*p*=0.002). One of the 10 teens obtained their license without having received a temporary permit prior to age 18. Although 9 teens obtained a prior temporary permit (mean age=16 years 3 months, SD=8 months), only 2 had a previous probationary license. Sex was not significantly associated with driving status.

**Table 2:** Driving Status of Teens Who Were Age-Eligible to Drive (Groups 2–4)

Driving status	Age group, *n* (%)		
	Group 2 (*n*=16)	Group 3 (*n*=14)	Group 4 (*n*=15)
Driving (total)	4 (25%)	5 (36%)	11 (73%)
Temporary permit	4	4	1
Probationary license	0	1	na^a^
Ohio driver’s license	na^a^	na^a^	10
Not driving	12 (75%)	9 (64%)	4 (27%)

*Note:*^a^In Ohio, all probationary licenses become unrestricted Ohio driver’s licenses when the driver turns age 18 years.

na, not applicable.

Fourteen of the 25 age-eligible teens (8 males, 54%) who were not driving were interested in obtaining a temporary permit. The remaining 11 (44%) were either uninterested or unsure. Reasons given by caregivers for disinterest in driving included having a disability that prevented the teen from driving (*n*=5), lack of teen confidence that they would be able to learn to drive or be good at driving (*n*=4), a physician’s recommendation that the teen was not ready (*n*=2), and caregiver preference that their teen delay driving (*n*=1), with some caregivers reporting multiple reasons for their teens’ disinterest. As summarized in Table 3, a significantly lower rate of driving in age-eligible teens was evident only when comparing teens with and without a neurological disorder (19% vs 59%).

**Table 3:** Frequencies (Percentages) of Physical and Developmental Disorders Among Teens Who Were Age-Eligible to Drive (Groups 2–4)

Disorder	Overall, *n* (%) (N=45)	Not driving, *n* (%) (N=25)	Driving, *n* (%) (N=20)	*p*-value^a^
Neurological				
Present	16 (36%)	13 (81%)	3 (19%)	**0.013**
Absent	29 (64%)	12 (41%)	17 (59%)	
Chronic, non-neurological				
Present	11 (24%)	6 (55%)	5 (45%)	0.271
Absent	34 (76%)	19 (56%)	15 (44%)	
Vision or hearing impairment				
Present	10 (22%)	6 (60%)	4 (40%)	0.748
Absent	35 (78%)	19 (54%)	16 (46%)	
Developmental				
Present	35 (78%)	21 (60%)	14 (40%)	0.301
Absent	10 (22%)	4 (40%)	6 (60%)	
Any of the above				
Present	39 (87%)	23 (59%)	16 (41%)	0.383
Absent	6 (13%)	2 (33%)	4 (67%)	

*Note:* Boldface indicates statistical significance (*p*<0.05).

a *p*-values were calculated with Fisher’s exact test for all comparisons.

Caregivers endorsed multiple concerns about teen driving. The frequency of these concerns is summarized in Table 4 for the teens in Group 1 and Groups 2–4. Common concerns were that the teen would (1) have difficulty noticing road hazards or adapting to changing road conditions, (2) have trouble responding to an unexpected event, and (3) lack the maturity needed to drive or would get lost. About half of the caregivers in Group 1 were concerned that their teen would not be skilled enough to drive safely even if they obtained their license, would go places without adult supervision, and would have difficulty learning how to drive or passing their driving test. Concerns that the teen lacked the maturity to drive or would get lost and that teens without a probationary or full license would have difficulty learning how to drive and passing the driving test were significantly more common for the 25 age-eligible teens who were not driving than for the 20 age-eligible teens who were driving. Consistent with this finding, caregivers of only 5 (25%) of the 20 driving teens were concerned that their teen lacked confidence in their driving skills or was less skilled than their peers.

**Table 4:** Caregiver Concerns

Concern	Age group, *n* (%)				*p*-value^a^
	Group 1	Groups 2–4			
	(*n*=15)	Total (*n*=45)	Not driving (*n*=25)	Driving (*n*=20)	
Teen will not be skilled enough to drive safely even if they get their license	7 (47%)	17 (38%)	11 (44%)	6 (30%)	0.336
Teen will have difficulty noticing road hazards or adapting to changing road conditions	12 (80%)	22 (49%)	13 (52%)	9 (45%)	0.641
Teen will have trouble responding to an unexpected event	11 (73%)	29 (64%)	16 (64%)	13 (65%)	0.945
Teen will go places without adult supervision	8 (53%)	7 (16%)	6 (24%)	1 (5%)	0.112
Teen will lack the maturity needed to drive or would get lost	7 (47%)	24 (53%)	17 (68%)	7 (35%)	**0.029**
Teen will have difficulty learning how to drive or passing their driving test^b^	8 (53%)	13 (38%)	12 (48%)	1 (5%)	**0.002**
Teen will not have a car to drive or way to cover the extra expense involved in allowing the teen to drive^c^	3 (20%)	9 (36%)	9 (36%)	—	—
Teen lacks confidence in their driving skills^d^	na	5 (25%)	—	5 (25%)	—
Teen is less skilled than other drivers their age^d^	na	5 (25%)	—	5 (25%)	—

*Note:* Boldface indicates statistical significance (*p*<0.05).

a *p*-value for differences between not driving and driving subgroups as calculated using chi-square or Fisher’s exact test.

b Concern was asked to caregivers of teens who were not yet driving independently without parent or adult supervision. This includes teens who were not yet age-eligible to drive and teens who were age-eligible but did not yet have a probationary or full driver’s license (*n*=15 in Group 1 and *n*=34 in Groups 2–4).

c Concern was asked to caregivers of teens who were not yet driving (*n*=15 in Group 1 and *n*=25 in Groups 2–4).

d Concern was asked to caregivers of teens who were driving (*n*=20 in Groups 2–4). na, not applicable.

The 9 caregivers of teens from Groups 2–4 who had a temporary permit assigned mean ratings in the *frequent* range (mean=4.1, SD=0.3) for talking to their teen about driving and in the *sometimes* range (mean=3.3, SD=0.2) for showing their teen how to drive. However, they only assigned a mean rating in the *rarely* range (mean=2.5, SD=0.3) for having their teen practice driving with them.

## DISCUSSION

Consistent with the high rate of neurobehavioral impairment in children born EP and the broad range of disorders listed in the survey, the majority (87%) of the present sample had parent-reported histories of physical or developmental disorders.^27^^,^^32^ Findings from the present survey support the hypothesis that there would be a high level of caregiver concern about the driving readiness of their teens. As documented in previous research on teen driving, common concerns were that the teens would have difficulties in handling unexpected events and noticing road hazards or adapting to changing road conditions.^6^, ^7^, ^8^, ^9^, ^10^, ^11^^,^^33^ Some concerns were more frequently endorsed for teens who were age-eligible but not driving than for teens who were driving, suggesting that teen characteristics are associated with failure to progress through the GDL process.

Caregiver concerns are justified by findings indicating that MVCs are the leading cause of death for U.S. teens, particularly in the early months of licensure primarily because of inexperience.^1^, ^2^, ^3^^,^^6^, ^7^, ^8^, ^9^, ^10^, ^11^^,^^33^ Teens with cognitive, physical, or visual impairments face an even higher crash risk.^15^, ^16^, ^17^, ^18^, ^19^, ^20^, ^21^, ^22^, ^23^, ^24^, ^25^ Problems in driving readiness have also been documented in teens with neurological and developmental disorders, including ADHD, ASD, and mood disorders.^16^, ^17^, ^18^, ^19^, ^20^, ^21^, ^22^, ^23^, ^24^, ^25^^,^^33^^,^^39^ Caregivers of teens born EP, like those of teens with these conditions, may choose to delay or restrict their teen’s driving training. However, postponing licensure may limit the benefits of GDL laws, which are designed to help new drivers gain experience under lower-risk conditions.^12^, ^13^, ^14^

Most (60%) of the teens born EP of prelicensure age were reported to have an interest in learning to drive. However, only a minority (30%) of the teens aged <18 years who were eligible for a temporary permit had obtained it, and only 1 of 14 teens aged 16–17 years (7%) had obtained an independent (probationary) license. The authors did not find any data on the percentage of age-eligible teens with temporary permits in the broader population. However, these findings suggest markedly lower rates of probationary licenses compared to results from a 2019 survey indicating that 60% of teens aged <18 years had a driver’s license (probationary or full).^40^ Although a majority (67%) of the teens aged 18 years in the study sample were licensed, 1 had never obtained a temporary permit, and only 2 obtained a probationary license prior to age 18 years. Results suggest that most teens born EP eventually become licensed drivers, but only few take full advantage of opportunities for supervised driving as part of the GLD process.

Among age-eligible teens born EP, rates of driving were lower for those with a physical or developmental disorder. The frequency of age-eligible teens who were driving was far lower for those with a neurological disorder compared to those without such a disorder (19% vs 59%). Prior research shows that teens born EP often face cognitive, learning, motor, and behavioral challenges—such as attention difficulties, coordination issues, impulsivity, or passivity—that affect planning, attention focus, and multitasking,^27^, ^28^, ^29^, ^30^ all of which may be associated with delays in driving readiness. Obtaining a driver’s license is key to fostering independence, allowing teens to access employment, education, and social activities.^3^ However, if a teen is not ready to drive, starting the process prematurely could have the opposite effect, including increasing risks for both the teen and other road users. In the case of other youth at high risk for developmental disabilities, support in the learning-to-drive process for teens born EP may require consultation with healthcare professionals to assess teen driving skills and potential challenges.^3^^,^^20^^,^^21^^,^^23^ If families decide to proceed with driving education, tailored training including extra practice, adaptive techniques, personalized feedback, and specialized instructors may be useful in helping these teens build the skills and confidence required for safe driving.^2^^,^^21^, ^22^, ^23^

Caregivers of teens with a temporary permit often talked and showed the teen how to drive, but were less likely to practice driving with their teen, a finding that is consistent with previous studies indicating that parents tend to play a more passive role in helping their teen become skilled drivers.^41^^,^^42^ More intensive on-road instructions may be especially important for teens with neurodevelopmental disabilities.^3^^,^^21^^,^^23^ Parents play a vital role in their teens’ driving experience by setting rules, teaching skills, and reinforcing safe driving behavior.^36^^,^^42^, ^43^, ^44^, ^45^, ^46^ Safe driving is best learned through supervised practice mandated under GDL laws in all 50 states in the U.S. and the District of Columbia, typically with a parent.^47^^,^^48^ Driving is a complex task requiring decision making and motor coordination; hence, hands-on practice is critical.^3^ Parents can use these sessions as teachable moments to build their teens driving skills and confidence. Teens born EP may also benefit from starting individualized driving supervision and practice earlier in the GLD process.

### Limitations

Limitations for this study include the lack of a control group of teens born full term, relatively small sample size, and recruitment of a single-hospital sample of caregivers of teens residing in Ohio. Potential self-selection biases related to the caregiver’s decision to participate further limit the generalizability of the findings to the broader population of teens born EP. An additional limitation is reliance on caregiver report for information on the teen’s driving status, the dates on which permits or licenses were obtained, and the teen’s history of physical and developmental conditions. Without having assessed the teens themselves, the authors cannot determine the extent to which caregiver perceptions reflect the teens’ interests and concerns. The caregiver survey also requires validation and potential expansion to include items that provide additional information on barriers to advancing toward licensure and reasons why parents rarely practice driving with their teens.

## CONCLUSIONS

Findings from this caregiver survey of driving readiness of teens (aged 14–18 years) born EP revealed that these teens frequently have neurodevelopmental or medical conditions and that only a minority of those aged <18 years who are eligible to drive obtain a learner’s permit. Caregivers voiced concerns about their teens’ abilities to notice road hazards, adapt to changing road conditions, and handle unexpected events while driving. These findings underscore the importance of tailored driving training to help teens born EP develop the skills and confidence needed to become safe drivers. The findings also support the appropriateness of consultation with healthcare providers and safety professionals to evaluate teen readiness to drive, including using objective driving assessments.^49^ Individualized training plans may also be useful in providing driver education. These plans might include starting supervised driving sessions earlier, extending supervised driving periods, and incorporating adaptive techniques and specialized instructors. Future research and policy should also consider longitudinal designs to examine progression toward licensure in this population and ways to facilitate entry of teens with developmental disabilities into the GDL process. Examples include studies to explore the benefits of providing more supervised practice for teens born EP, using technology (e.g., driving simulators and DriveCam) for tailored training to support parents in teaching driving skills, and examining the utility and acceptability of virtual or driving assist systems to improve driving safety of teens born EP.^50^, ^51^, ^52^, ^53^, ^54^
